# *e*,*e*-*trans*-Cyclo­hexane-1,4-carb­oxy­lic acid–hexa­methyl­ene­tetra­mine (1/2)

**DOI:** 10.1107/S1600536810053626

**Published:** 2011-01-08

**Authors:** Andreas Lemmerer

**Affiliations:** a Molecular Sciences Institute, School of Chemistry, University of the Witwatersrand, Private Bag 3, PO WITS, 2050, Johannesburg, South Africa

## Abstract

The asymmetric unit of the title compound, 2C_6_H_12_N_4_·C_8_H_12_O_4_, contains one half-mol­ecule of *e*,*e*-*trans*-cyclo­hexane-1,4-dicarb­oxy­lic acid (the complete molecule being generated by inversion symmetry) and one mol­ecule of hexa­methyl­ene­tetra­mine (HMTA), which are connected by O—H⋯N hydrogen bonds. This forms isolated trimers that pack in a herringbone fashion.

## Related literature

For related co-crystals featuring one hydrogen bond to HMTA, see: Feng *et al.* (2006 ▶); Li *et al.* (2001 ▶); Mak *et al.* (1986 ▶). For related co-crystals featuring two hydrogen bonds to HMTA, see: Coupar *et al.* (1997*a* ▶); Gardon *et al.* (2003 ▶); Ghosh *et al.* (2005 ▶). For related co-crystals featuring three hydrogen bonds to HMTA, see: Coupar *et al.* (1997*b* ▶); De Bruyn *et al.* (1996 ▶); Jordan & Mak (1970 ▶). For related co-crystals featuring four hydrogen bonds to HMTA, see: Daka & Wheeler (2006 ▶); MacLean *et al.* (1999 ▶); Zakaria *et al.* (2003 ▶).
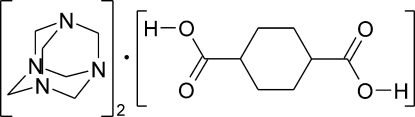


## Experimental

### 

#### Crystal data

2C_6_H_12_N_4_·C_8_H_12_O_4_
*M* *_r_* = 452.57
Monoclinic, 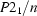

*a* = 5.9182 (1) Å
*b* = 31.5242 (6) Å
*c* = 6.1193 (1) Å
β = 109.144 (1)°
*V* = 1078.52 (3) Å^3^
*Z* = 2
Mo *K*α radiation
μ = 0.1 mm^−1^
*T* = 173 K
0.4 × 0.35 × 0.04 mm

#### Data collection

Bruker APEXII CCD area-detector diffractometer
Absorption correction: integration (*XPREP*; Bruker, 2004 ▶) *T*_min_ = 0.960, *T*_max_ = 0.996
13851 measured reflections
2594 independent reflections
2218 reflections with *I* > 2σ(*I*)
*R*_int_ = 0.045

#### Refinement

*R*[*F* ^2^ > 2σ(*F* ^2^)] = 0.040
*wR*(*F* ^2^) = 0.113
*S* = 0.89
2594 reflections
146 parameters
H-atom parameters constrained
Δρ_max_ = 0.39 e Å^−3^
Δρ_min_ = −0.19 e Å^−3^

### 

Data collection: *APEX2* (Bruker, 2005 ▶); cell refinement: *SAINT-Plus* (Bruker, 2004 ▶); data reduction: *SAINT-Plus* and *XPREP* (Bruker 2004 ▶); program(s) used to solve structure: *SHELXS97* (Sheldrick, 2008 ▶); program(s) used to refine structure: *SHELXL97* (Sheldrick, 2008 ▶); molecular graphics: *ORTEP-3 for Windows* (Farrugia, 1997 ▶) and *DIAMOND* (Brandenburg, 1999 ▶); software used to prepare material for publication: *WinGX* (Farrugia, 1999 ▶) and *PLATON* (Spek, 2009 ▶).

## Supplementary Material

Crystal structure: contains datablocks global, I. DOI: 10.1107/S1600536810053626/bt5446sup1.cif

Structure factors: contains datablocks I. DOI: 10.1107/S1600536810053626/bt5446Isup2.hkl

Additional supplementary materials:  crystallographic information; 3D view; checkCIF report

## Figures and Tables

**Table 1:** Hydrogen-bond geometry (Å, °)

*D*—H⋯*A*	*D*—H	H⋯*A*	*D*⋯*A*	*D*—H⋯*A*
O1—H1⋯N1	0.84	1.84	2.6710 (12)	170

**Fig. 1.**
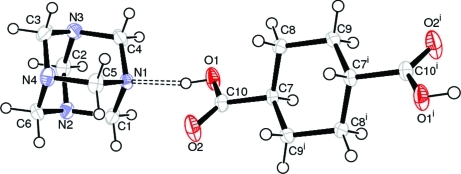
View of (I) (50% probability displacement ellipsoids). Symmetry code: (i):2 - x, 1 - y, 2 - z.

**Fig. 2.**
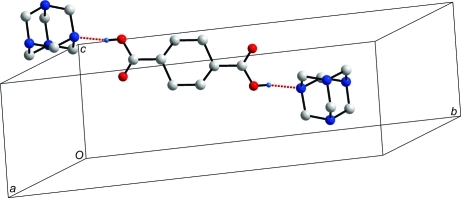
Hydrogen bonded centrosymmetric trimers formed by simple hydrogen bonds (red dashed lines) between one CHDA and two HMTA molecules.

**Fig. 3.**
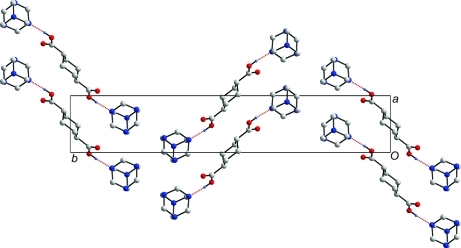
Packing diagram of the trimers viewed down the c axis.

## supplementary crystallographic information

### Comment

The molecule hexamethylenetetramine (HMTA) acts as a good hydrogen bond acceptor for carboxylic acid or phenol hydrogen bond donors to make binary co-crystals. HMTA can accept any number from one to four hydrogen bonds due to its tetravalent hydrogen bond acceptors, the lone pairs on each of the four N atoms. Most common are two hydrogen bonds per HMTA molecule (Coupar *et al.*, 1997*a*; Gardon *et al.*, 2003; Ghosh *et al.*, 2005.), with a smaller frequency of one, three or four hydrogen bonds (Coupar *et al.*, 1997*b*; Daka & Wheeler, 2006; De Bruyn *et al.*, 1996; Feng *et al.*, 2006; Jordan & Mak, 1970; Li *et al.*, 2001; MacLean *et al.*, 1999; Mak *et al.*, 1986; Zakaria *et al.*, 2003.). The title compound is an example of HMTA only accepting one hydrogen bond. As the *cis*-cyclohexane-1,4-dicarboxylic acid (CHDA) molecule has two carboxylic acid groups, the observed hydrogen bonded assembly is dumb-bell shaped, where one central CHDA molecule (the bar) hydrogen bonds to two pendant HMTA molecules (the bells) (Fig. 2). The dumb-bell trimers are situated on a centre of inversion, located at the centre of the CHDA molecule. The dumbells pack in a herring-bone fashion (Fig. 3).

### Experimental

Crystals where grown by slow evaporation at ambient conditions of a methanol solution containing a 2:1 stoichiometric quantity of hexamethylenetetramine and *cis*-1,4-cyclohexanecarboxylic acid.

### Refinement

The C-bound H atoms were geometrically placed with C—H bond lengths of 1.00Å for methine CH and 0.99Å for ethylene CH_2_ and refined as riding with *U*_iso_(H) = 1.2*U*_eq_(C). The O-bound H atom was geometrically placed (O—H bond length 0.84 Å) and refined as riding with *U*_iso_(H) = 1.5*U*_eq_(O).

### Crystal data

**Table:**

2C_6_H_12_N_4_·C_8_H_12_O_4_	*F*(000) = 488
*M**_r_* = 452.57	*D*_x_ = 1.394 Mg m^−^^3^
Monoclinic, *P*2_1_/*n*	Mo *K*α radiation, λ = 0.71073 Å
Hall symbol: -P 2yn	Cell parameters from 5661 reflections
*a* = 5.9182 (1) Å	θ = 3.7–28.3°
*b* = 31.5242 (6) Å	µ = 0.1 mm^−^^1^
*c* = 6.1193 (1) Å	*T* = 173 K
β = 109.144 (1)°	Plate, colourless
*V* = 1078.52 (3) Å^3^	0.4 × 0.35 × 0.04 mm
*Z* = 2	

### Data collection

**Table:**

Bruker APEXII CCD area-detector diffractometer	2218 reflections with *I* > 2σ(*I*)
ω scans	*R*_int_ = 0.045
Absorption correction: integration (*XPREP*; Bruker, 2004)	θ_max_ = 28.0°, θ_min_ = 1.3°
*T*_min_ = 0.960, *T*_max_ = 0.996	*h* = −7→7
13851 measured reflections	*k* = −41→41
2594 independent reflections	*l* = −8→8

### Refinement

**Table:**

Refinement on *F*^2^	0 restraints
Least-squares matrix: full	H-atom parameters constrained
*R*[*F*^2^ > 2σ(*F*^2^)] = 0.040	*w* = 1/[σ^2^(*F*_o_^2^) + (0.0739*P*)^2^ + 0.3667*P*] where *P* = (*F*_o_^2^ + 2*F*_c_^2^)/3
*wR*(*F*^2^) = 0.113	(Δ/σ)_max_ = 0.001
*S* = 0.89	Δρ_max_ = 0.39 e Å^−^^3^
2594 reflections	Δρ_min_ = −0.19 e Å^−^^3^
146 parameters	

### Special details

**Table:**

Experimental. Numerical integration absorption corrections based on indexed crystal faces were applied using the *XPREP* routine (Bruker, 2004)
Geometry. All e.s.d.'s (except the e.s.d. in the dihedral angle between two l.s. planes) are estimated using the full covariance matrix. The cell e.s.d.'s are taken into account individually in the estimation of e.s.d.'s in distances, angles and torsion angles; correlations between e.s.d.'s in cell parameters are only used when they are defined by crystal symmetry. An approximate (isotropic) treatment of cell e.s.d.'s is used for estimating e.s.d.'s involving l.s. planes.

### Fractional atomic coordinates and isotropic or equivalent isotropic displacement parameters (Å^2^)

**Table:**

	*x*	*y*	*z*	*U*_iso_*/*U*_eq_	
C1	−0.0247 (2)	0.63264 (4)	0.4650 (2)	0.0246 (3)	
H1A	−0.1214	0.6094	0.3707	0.029*	
H1B	−0.0231	0.6289	0.6261	0.029*	
C2	−0.1335 (2)	0.67841 (4)	0.1382 (2)	0.0282 (3)	
H2A	−0.2086	0.7058	0.0755	0.034*	
H2B	−0.2303	0.6555	0.041	0.034*	
C3	0.2502 (2)	0.71082 (4)	0.2722 (2)	0.0290 (3)	
H3A	0.1799	0.7386	0.2111	0.035*	
H3B	0.4155	0.7101	0.2667	0.035*	
C4	0.2168 (2)	0.63619 (4)	0.2177 (2)	0.0282 (3)	
H4A	0.3818	0.6348	0.2117	0.034*	
H4B	0.1235	0.613	0.1201	0.034*	
C5	0.3614 (2)	0.66535 (4)	0.6002 (2)	0.0256 (3)	
H5A	0.3658	0.662	0.7623	0.031*	
H5B	0.5281	0.6642	0.5986	0.031*	
C6	0.0111 (2)	0.70746 (4)	0.5164 (2)	0.0267 (3)	
H6A	0.0126	0.7045	0.678	0.032*	
H6B	−0.0621	0.7352	0.4574	0.032*	
N1	0.22317 (16)	0.62980 (3)	0.45945 (16)	0.0202 (2)	
N2	−0.13588 (17)	0.67350 (3)	0.37597 (19)	0.0250 (2)	
N3	0.1098 (2)	0.67701 (3)	0.12396 (18)	0.0273 (2)	
N4	0.25799 (18)	0.70679 (3)	0.51366 (18)	0.0257 (2)	
C7	0.76647 (19)	0.52006 (3)	0.88313 (19)	0.0188 (2)	
H7	0.6734	0.4942	0.8128	0.023*	
C8	0.9750 (2)	0.52451 (4)	0.78814 (19)	0.0219 (2)	
H8A	0.9119	0.5271	0.6175	0.026*	
H8B	1.0667	0.5506	0.851	0.026*	
C9	1.1394 (2)	0.48596 (4)	0.85539 (19)	0.0213 (2)	
H9A	1.0504	0.4603	0.7819	0.026*	
H9B	1.2758	0.4897	0.7973	0.026*	
C10	0.6008 (2)	0.55801 (3)	0.8143 (2)	0.0212 (2)	
O1	0.47562 (17)	0.55861 (3)	0.59260 (15)	0.0319 (2)	
H1	0.3883	0.5803	0.5629	0.048*	
O2	0.58343 (19)	0.58498 (3)	0.94835 (17)	0.0397 (3)	

### Atomic displacement parameters (Å^2^)

**Table:**

	*U*^11^	*U*^22^	*U*^33^	*U*^12^	*U*^13^	*U*^23^
C1	0.0217 (5)	0.0197 (6)	0.0322 (6)	0.0004 (4)	0.0087 (5)	0.0055 (4)
C2	0.0237 (6)	0.0261 (6)	0.0265 (6)	0.0051 (5)	−0.0031 (5)	0.0042 (5)
C3	0.0264 (6)	0.0249 (6)	0.0384 (7)	0.0007 (5)	0.0145 (5)	0.0100 (5)
C4	0.0356 (7)	0.0266 (6)	0.0238 (6)	0.0119 (5)	0.0115 (5)	0.0007 (5)
C5	0.0203 (5)	0.0246 (6)	0.0253 (6)	0.0009 (4)	−0.0015 (4)	0.0010 (4)
C6	0.0330 (6)	0.0203 (6)	0.0297 (6)	0.0059 (5)	0.0143 (5)	−0.0018 (4)
N1	0.0193 (5)	0.0187 (5)	0.0205 (5)	0.0044 (3)	0.0038 (4)	0.0017 (3)
N2	0.0178 (5)	0.0221 (5)	0.0353 (6)	0.0038 (4)	0.0091 (4)	0.0049 (4)
N3	0.0350 (6)	0.0273 (6)	0.0206 (5)	0.0083 (4)	0.0104 (4)	0.0052 (4)
N4	0.0247 (5)	0.0190 (5)	0.0296 (5)	−0.0022 (4)	0.0037 (4)	−0.0008 (4)
C7	0.0179 (5)	0.0154 (5)	0.0212 (5)	0.0034 (4)	0.0039 (4)	0.0004 (4)
C8	0.0242 (6)	0.0216 (5)	0.0206 (5)	0.0059 (4)	0.0084 (4)	0.0050 (4)
C9	0.0216 (5)	0.0220 (6)	0.0218 (5)	0.0056 (4)	0.0090 (4)	0.0026 (4)
C10	0.0183 (5)	0.0181 (5)	0.0246 (6)	0.0025 (4)	0.0037 (4)	0.0006 (4)
O1	0.0369 (5)	0.0259 (5)	0.0249 (5)	0.0161 (4)	−0.0007 (4)	−0.0002 (3)
O2	0.0487 (6)	0.0305 (5)	0.0306 (5)	0.0201 (4)	0.0002 (4)	−0.0064 (4)

### Geometric parameters (Å, °)

**Table:**

C1—N2	1.4676 (14)	C5—H5B	0.99
C1—N1	1.4808 (15)	C6—N2	1.4663 (16)
C1—H1A	0.99	C6—N4	1.4671 (16)
C1—H1B	0.99	C6—H6A	0.99
C2—N2	1.4678 (17)	C6—H6B	0.99
C2—N3	1.4713 (17)	C7—C10	1.5160 (15)
C2—H2A	0.99	C7—C9^i^	1.5238 (15)
C2—H2B	0.99	C7—C8	1.5331 (15)
C3—N3	1.4679 (17)	C7—H7	1
C3—N4	1.4686 (16)	C8—C9	1.5268 (15)
C3—H3A	0.99	C8—H8A	0.99
C3—H3B	0.99	C8—H8B	0.99
C4—N3	1.4657 (15)	C9—C7^i^	1.5238 (15)
C4—N1	1.4812 (15)	C9—H9A	0.99
C4—H4A	0.99	C9—H9B	0.99
C4—H4B	0.99	C10—O2	1.2091 (15)
C5—N4	1.4662 (15)	C10—O1	1.3154 (14)
C5—N1	1.4848 (15)	O1—H1	0.84
C5—H5A	0.99		
			
N2—C1—N1	111.82 (9)	H6A—C6—H6B	107.8
N2—C1—H1A	109.3	C1—N1—C4	108.27 (9)
N1—C1—H1A	109.3	C1—N1—C5	107.61 (9)
N2—C1—H1B	109.3	C4—N1—C5	107.78 (9)
N1—C1—H1B	109.3	C6—N2—C1	108.39 (9)
H1A—C1—H1B	107.9	C6—N2—C2	107.89 (9)
N2—C2—N3	112.54 (9)	C1—N2—C2	108.14 (9)
N2—C2—H2A	109.1	C4—N3—C3	108.13 (10)
N3—C2—H2A	109.1	C4—N3—C2	107.97 (10)
N2—C2—H2B	109.1	C3—N3—C2	107.96 (9)
N3—C2—H2B	109.1	C5—N4—C6	107.94 (9)
H2A—C2—H2B	107.8	C5—N4—C3	108.17 (10)
N3—C3—N4	112.56 (9)	C6—N4—C3	107.91 (9)
N3—C3—H3A	109.1	C10—C7—C9^i^	111.75 (9)
N4—C3—H3A	109.1	C10—C7—C8	110.48 (9)
N3—C3—H3B	109.1	C9^i^—C7—C8	110.30 (9)
N4—C3—H3B	109.1	C10—C7—H7	108.1
H3A—C3—H3B	107.8	C9^i^—C7—H7	108.1
N3—C4—N1	112.12 (9)	C8—C7—H7	108.1
N3—C4—H4A	109.2	C9—C8—C7	110.25 (9)
N1—C4—H4A	109.2	C9—C8—H8A	109.6
N3—C4—H4B	109.2	C7—C8—H8A	109.6
N1—C4—H4B	109.2	C9—C8—H8B	109.6
H4A—C4—H4B	107.9	C7—C8—H8B	109.6
N4—C5—N1	112.20 (9)	H8A—C8—H8B	108.1
N4—C5—H5A	109.2	C7^i^—C9—C8	111.31 (9)
N1—C5—H5A	109.2	C7^i^—C9—H9A	109.4
N4—C5—H5B	109.2	C8—C9—H9A	109.4
N1—C5—H5B	109.2	C7^i^—C9—H9B	109.4
H5A—C5—H5B	107.9	C8—C9—H9B	109.4
N2—C6—N4	112.61 (9)	H9A—C9—H9B	108
N2—C6—H6A	109.1	O2—C10—O1	123.03 (11)
N4—C6—H6A	109.1	O2—C10—C7	123.88 (10)
N2—C6—H6B	109.1	O1—C10—C7	113.09 (10)
N4—C6—H6B	109.1	C10—O1—H1	109.5
			
N2—C1—N1—C4	−57.93 (12)	N2—C2—N3—C4	58.78 (12)
N2—C1—N1—C5	58.31 (12)	N2—C2—N3—C3	−57.91 (12)
N3—C4—N1—C1	57.99 (13)	N1—C5—N4—C6	58.47 (13)
N3—C4—N1—C5	−58.15 (13)	N1—C5—N4—C3	−58.05 (13)
N4—C5—N1—C1	−58.63 (12)	N2—C6—N4—C5	−58.39 (12)
N4—C5—N1—C4	57.94 (12)	N2—C6—N4—C3	58.29 (12)
N4—C6—N2—C1	58.57 (13)	N3—C3—N4—C5	58.48 (12)
N4—C6—N2—C2	−58.32 (12)	N3—C3—N4—C6	−58.06 (12)
N1—C1—N2—C6	−58.43 (13)	C10—C7—C8—C9	−179.28 (9)
N1—C1—N2—C2	58.29 (12)	C9^i^—C7—C8—C9	56.67 (13)
N3—C2—N2—C6	58.06 (12)	C7—C8—C9—C7^i^	−57.25 (13)
N3—C2—N2—C1	−58.99 (12)	C9^i^—C7—C10—O2	13.98 (17)
N1—C4—N3—C3	58.54 (13)	C8—C7—C10—O2	−109.24 (14)
N1—C4—N3—C2	−58.04 (13)	C9^i^—C7—C10—O1	−165.62 (10)
N4—C3—N3—C4	−58.70 (13)	C8—C7—C10—O1	71.17 (12)
N4—C3—N3—C2	57.88 (12)		

Symmetry codes: (i) −*x*+2, −*y*+1, −*z*+2.

### Hydrogen-bond geometry (Å, °)

**Table:**

*D*—H···*A*	*D*—H	H···*A*	*D*···*A*	*D*—H···*A*
O1—H1···N1	0.84	1.84	2.6710 (12)	170
